# Supplementary material for: Understanding the Structural and Catalytic Properties of Al(IV)-2 Acidic Sites of ZSM-5
Source: Materials (Basel). 2024 Jun 10;17(12):2824. doi: 10.3390/ma17122824 (PMC11204418; doi:10.3390/ma17122824)
Supplement: Supplementary file 1 [file materials-17-02824-s001.zip › materials-3034861-supplementary.pdf]

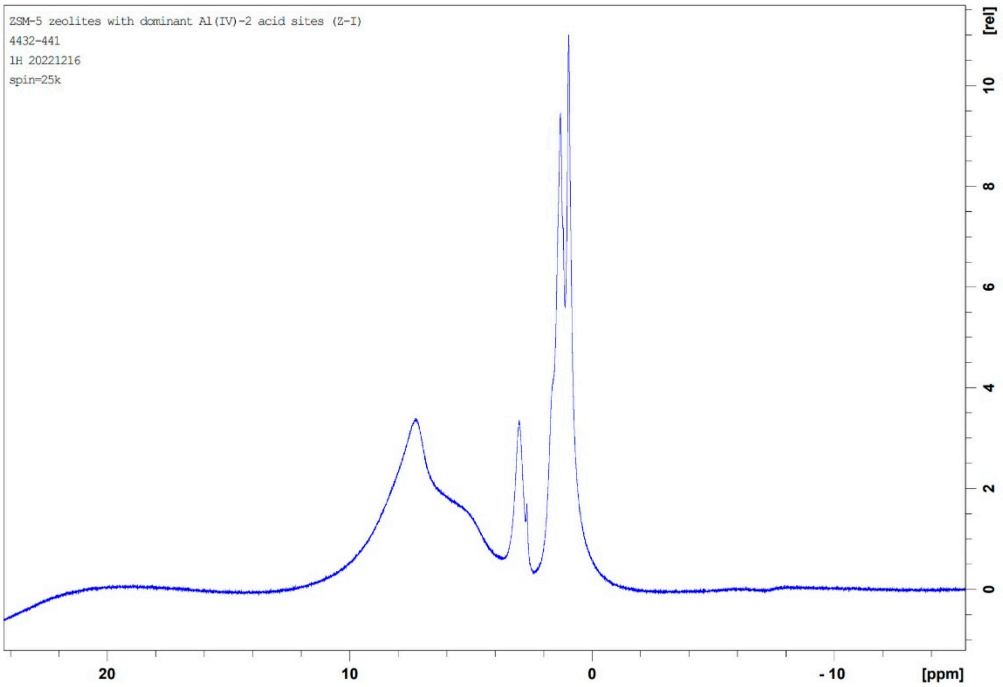

Figure S1. ^{1}H MAS NMR spectrum of Z-I.

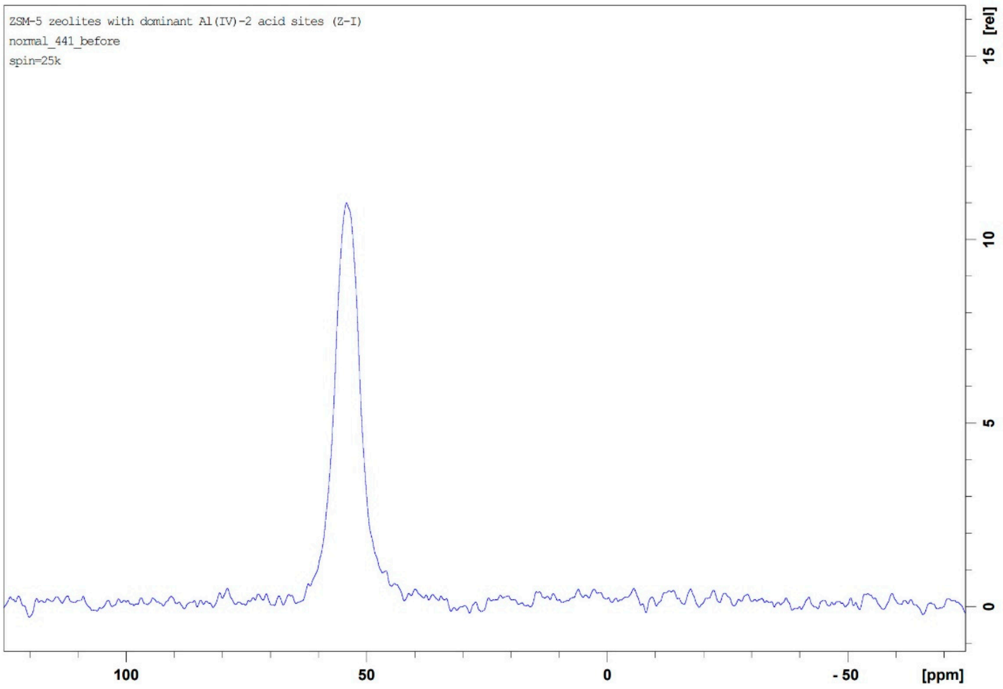

Figure S2. ^{27}Al MAS NMR spectrum of Z-I.

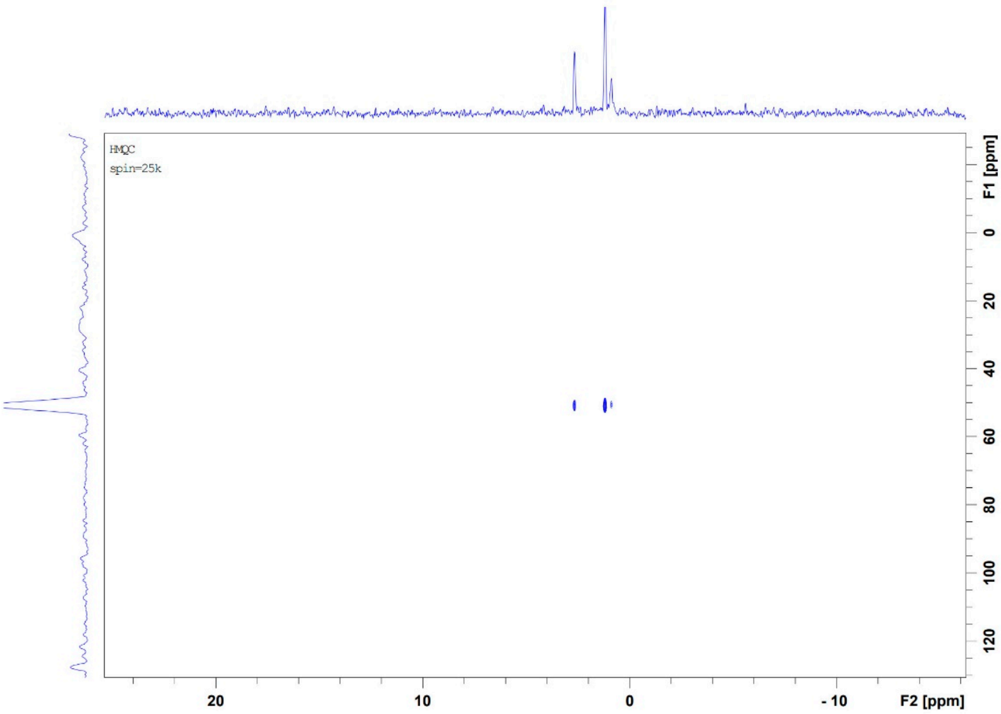

Figure S3. ^{1}H -^{27}Al D-HMQC 2D MAS NMR spectrum of Z-I.

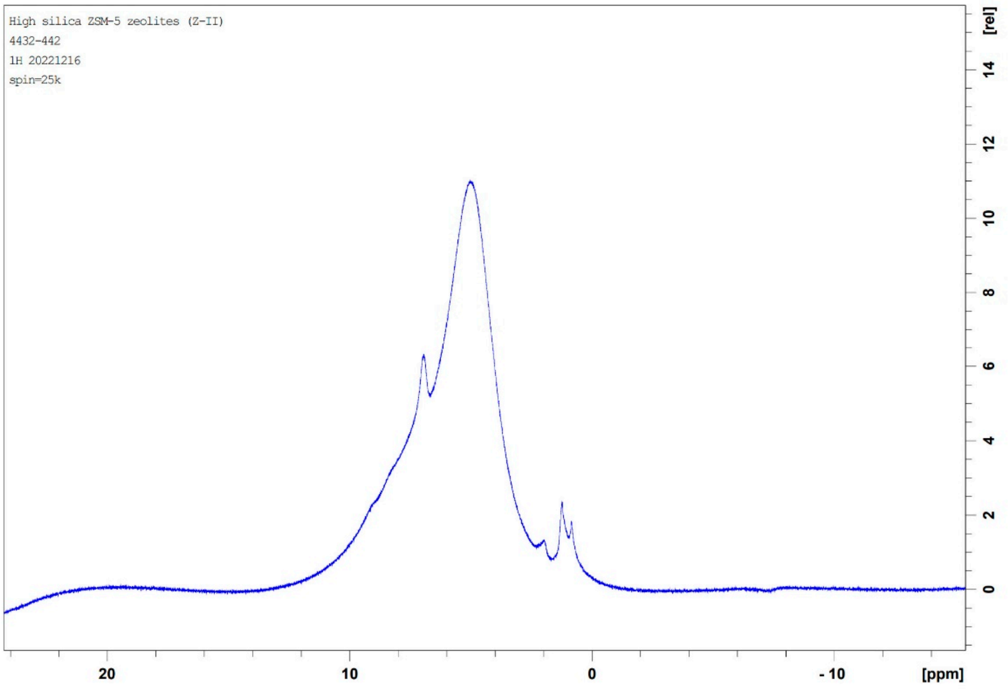

Figure S4. ^{1}H MAS NMR spectrum of Z-II.

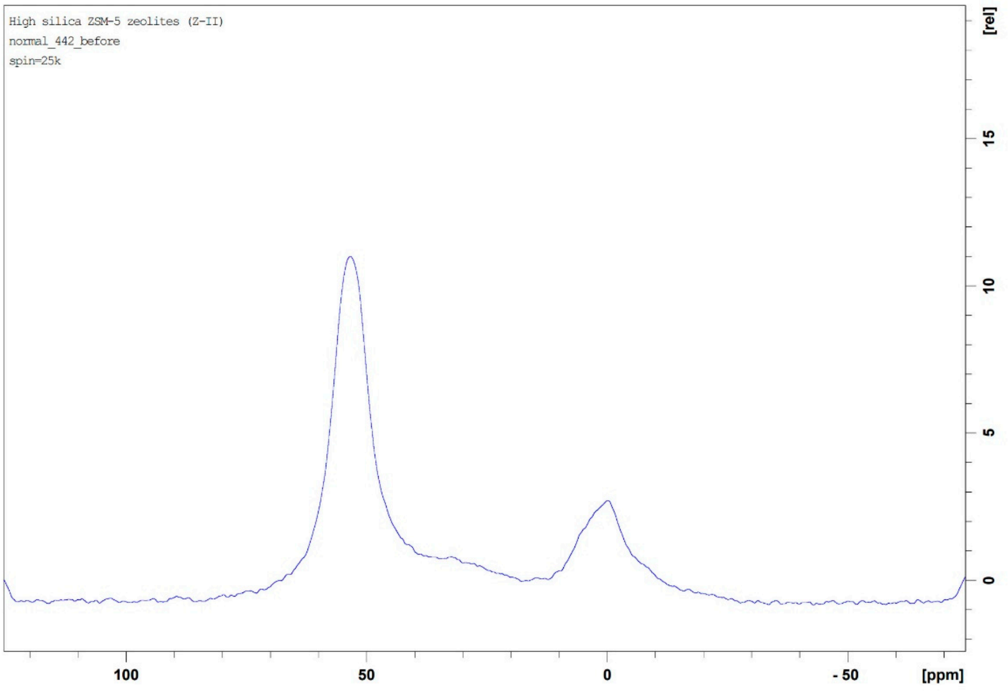

Figure S5. ^{27}Al MAS NMR spectrum of Z-II.

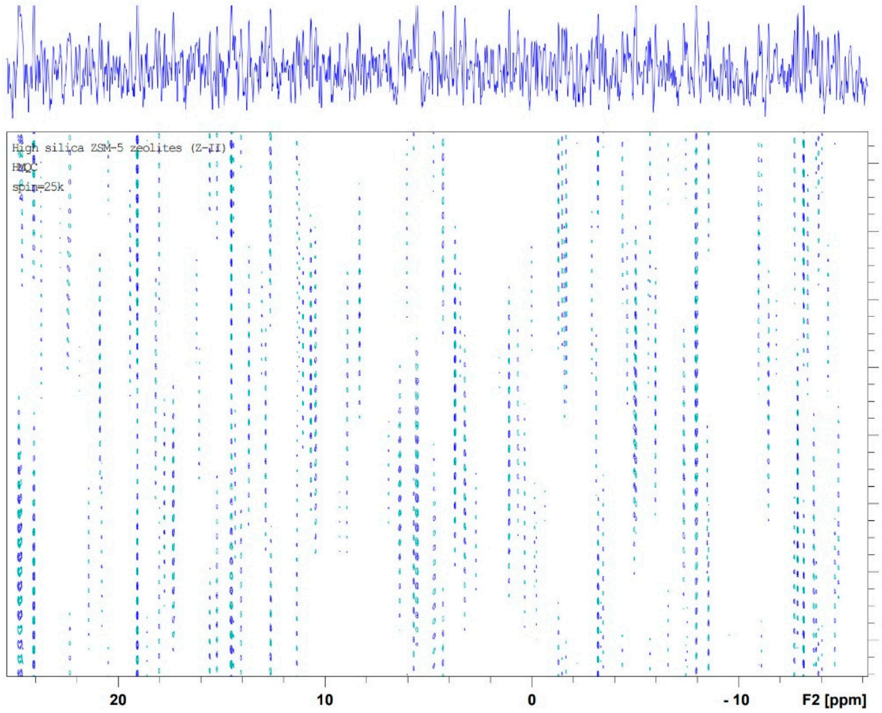

Figure S6. ^{1}H -^{27}Al D-HMQC 2D MAS NMR spectrum of Z-II.

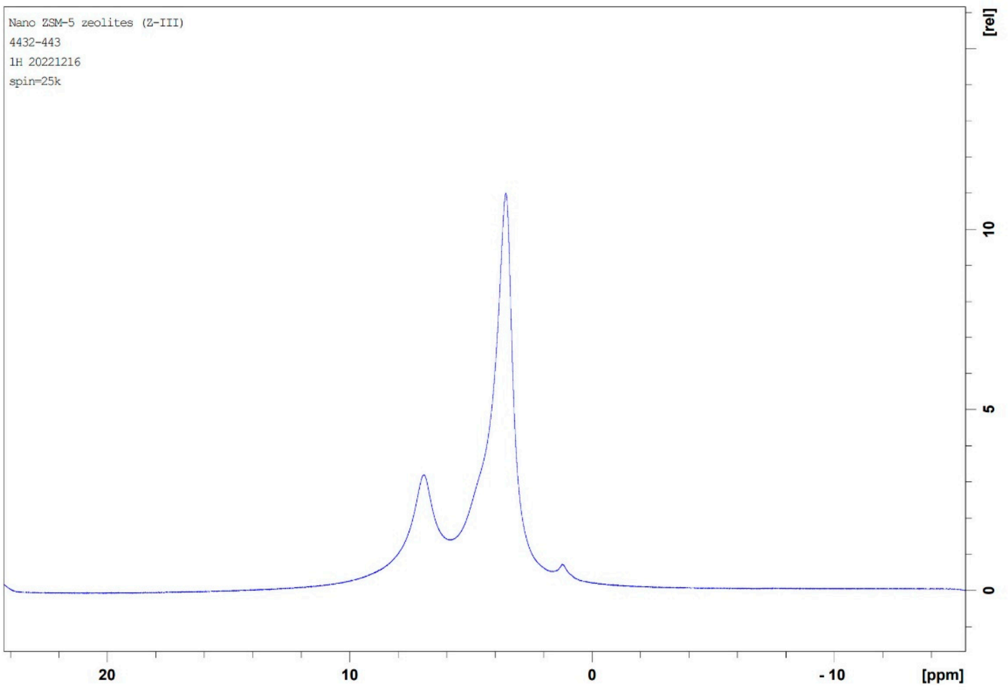

Figure S7. ^{1}H MAS NMR spectrum of Z-III.

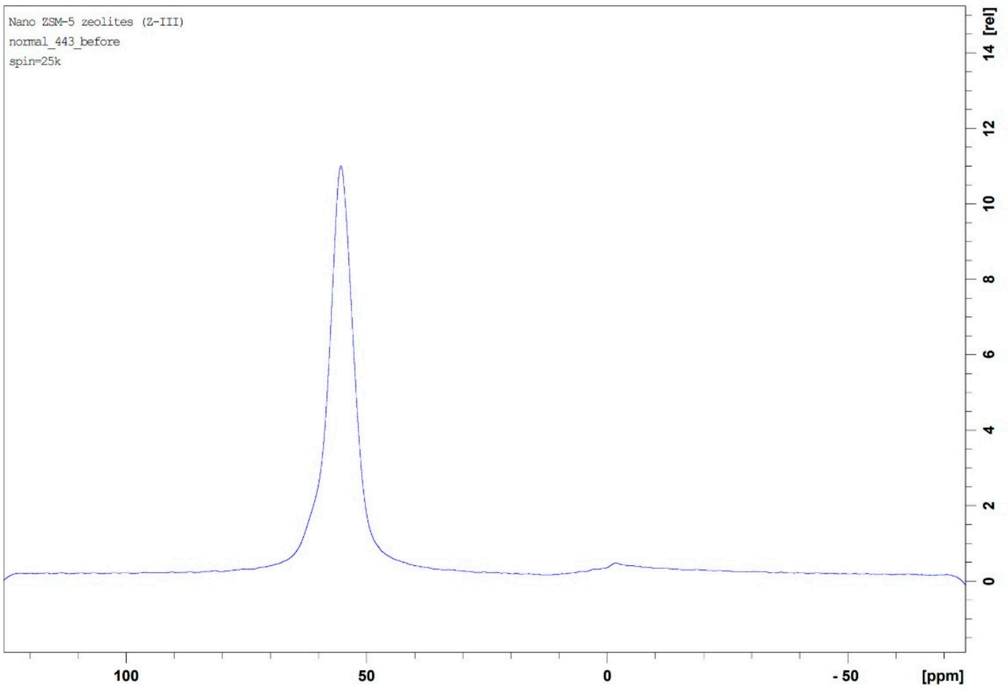

Figure S8. ^{27}Al MAS NMR spectrum of Z-III.

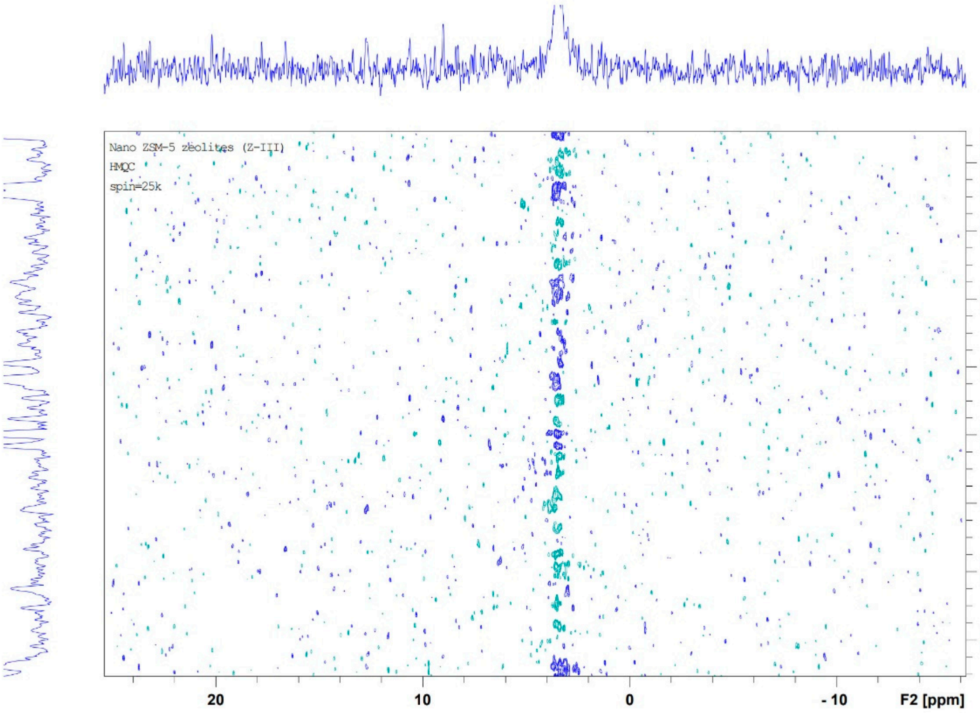

Figure S9. ^{1}H -^{27}Al D-HMQC 2D MAS NMR spectrum of Z-III.
